# A High-Resolution Map of Segmental DNA Copy Number Variation in the Mouse Genome

**DOI:** 10.1371/journal.pgen.0030003

**Published:** 2007-01-05

**Authors:** Timothy A Graubert, Patrick Cahan, Deepa Edwin, Rebecca R Selzer, Todd A Richmond, Peggy S Eis, William D Shannon, Xia Li, Howard L McLeod, James M Cheverud, Timothy J Ley

**Affiliations:** 1 Department of Medicine, Division of Oncology, Stem Cell Biology Section, Washington University, St. Louis, Missouri, United States of America; 2 NimbleGen Systems, Inc., Madison, Wisconsin, United States of America; 3 Department of Medicine, Division of General Medical Science, Washington University, St. Louis, Missouri, United States of America; 4 Department of Medicine, Division of Oncology, Molecular Oncology Section, Washington University, St. Louis, Missouri, United States of America; 5 Department of Anatomy and Neurobiology, Washington University, St. Louis, Missouri, United States of America; Harvard Medical School, United States of America

## Abstract

Submicroscopic (less than 2 Mb) segmental DNA copy number changes are a recently recognized source of genetic variability between individuals. The biological consequences of copy number variants (CNVs) are largely undefined. In some cases, CNVs that cause gene dosage effects have been implicated in phenotypic variation. CNVs have been detected in diverse species, including mice and humans*.* Published studies in mice have been limited by resolution and strain selection. We chose to study 21 well-characterized inbred mouse strains that are the focus of an international effort to measure, catalog, and disseminate phenotype data. We performed comparative genomic hybridization using long oligomer arrays to characterize CNVs in these strains. This technique increased the resolution of CNV detection by more than an order of magnitude over previous methodologies. The CNVs range in size from 21 to 2,002 kb. Clustering strains by CNV profile recapitulates aspects of the known ancestry of these strains. Most of the CNVs (77.5%) contain annotated genes, and many (47.5%) colocalize with previously mapped segmental duplications in the mouse genome. We demonstrate that this technique can identify copy number differences associated with known polymorphic traits. The phenotype of previously uncharacterized strains can be predicted based on their copy number at these loci. Annotation of CNVs in the mouse genome combined with sequence-based analysis provides an important resource that will help define the genetic basis of complex traits.

## Author Summary

A major goal of genetics and genomics is to understand how genetic differences between individuals (genotypes) translate into variation in disease susceptibility, behavior, and many other organism-level characteristics (phenotypes). While the sizes of genetic variants range from a single base to whole chromosomes, historically, only the extreme ends of this spectrum have been explored. DNA copy number variants (CNVs) lie between these two extremes, ranging in size from hundreds to millions of bases. The recent application of microarray technology to detect genetic variation in humans has led to the realization that CNVs are common. In fact, rough estimates indicate that CNVs and small-scale variants may constitute similar proportions of total genomic DNA. In this report, the authors characterize 80 CNVs across the genomes of 21 inbred strains of mice. The identification and characterization of mouse CNVs are important because inbred strains of mice are the most widely used model system to explore biomedical genetics. These CNVs are located near another class of genomic features, segmental duplications, more often than would be expected by chance, which supports the hypothesis that CNVs and segmental duplications are causally linked. Importantly, many of the CNVs contain known genes and thus may underlie both gene expression and phenotypic variation between strains.

## Introduction

Inbred mice are the model organisms of choice for studying the genetic basis of complex traits such as diabetes, heart disease, and cancer. A large and diverse set of traits has been systematically organized in the publicly accessible Mouse Phenome Database (MPD; www.jax.org/phenome), housed at The Jackson Laboratory (Bar Harbor, Maine, United States). The priority strains designated by the MPD serve as a standard set chosen by the genetics community to represent commonly used and genetically disparate inbred strains of Mus musculus and wild-derived subspecies. Current efforts are directed at identifying the full complement of genetic variants that influence heritable traits.

Sequence-based studies have begun to define the genetic differences that exist between these strains at the nucleotide level. The prevailing model is that the mouse genome is a mosaic of sequence blocks derived from ancestral populations, reflecting the unique breeding history of each strain [1,2]. Recently, variation in segmental DNA copy number has emerged as an additional dimension of genetic diversity that exists in the germline of rodents and primates. Copy number variants (CNVs) have been detected in humans, chimpanzees, and mice [3–7]. Germline CNVs affecting gene expression or function could contribute to heritable differences for many traits. More than 3,800 human CNVs have been identified and cataloged (http://projects.tcag.ca/variation). These CNVs extend the spectrum of genetic changes that contribute to phenotypic differences in mammalian species. In addition to effects on normal traits, CNVs are also likely to influence disease susceptibility [8]. Published studies of CNVs in mice have been limited by resolution and strain selection [6,7]. The aim of this study was to define the extent of CNV in the genomes of 21 well-characterized MPD strains. Using a high-density, tiling-path whole genome-long oligomer array, we identified CNVs in all strains and found that most of these segments contain annotated genes. The CNV profiles of these strains recapitulate aspects of their known ancestry. This high-resolution map of mouse CNVs in diverse strains will facilitate ongoing efforts to map phenotypes to genes.

## Results/Discussion

### High-Resolution Comparative Genomic Hybridization Analysis of MPD Strains

**Figure 1:**
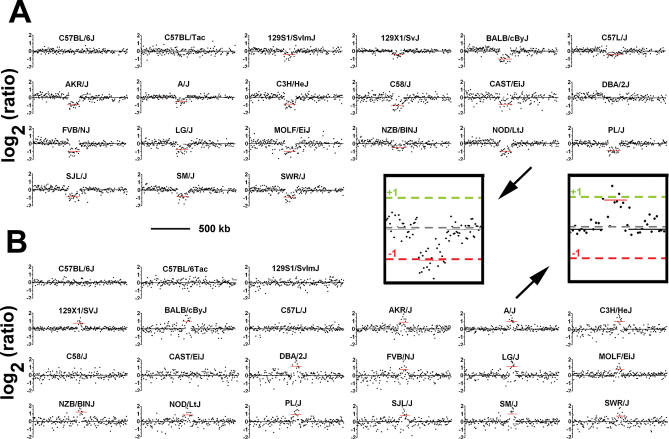
Representative Germline CNVs in Mice Identified by High-Resolution aCGH The log_2_ ratios of signal intensity for C57BL/6J (reference) versus 20 test strains are shown. Inset, an expanded view of the CNVs in NOD/LtJ and A/J from (A) and (B). Scale, 500 kb. (A) A 135.6-kb segment of reduced copy number (mean log_2_ = −1.02) on Chromosome 14 is present in most strains. (B) A 61.7-kb amplified segment (mean log_2_ = +1.01) on Chromosome 1 is present in most strains.

**Figure 2:**
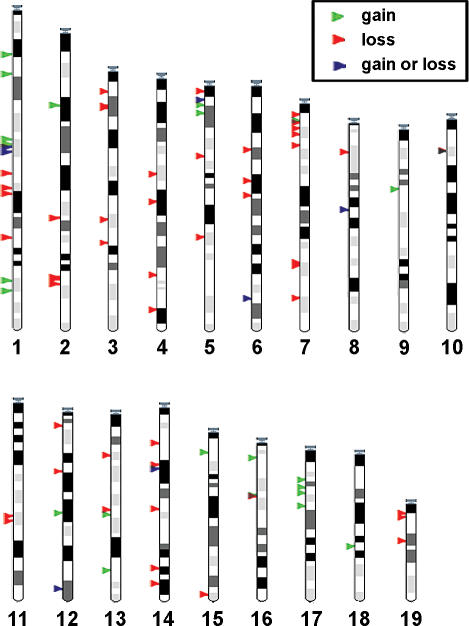
Genome-wide Distribution of CNVs The ideograms depict chromosomal locations of copy number gains (green arrows), losses (red arrows), and gains or losses (blue arrows) relative to C57BL/6J in autosomes from 20 inbred strains.

We performed comparative genomic hybridization using long oligonucleotide arrays (oligo-aCGH) containing 388,352 probes spanning the mouse reference genome with a median spacing of 5 kb. Germline DNA from 20 high-priority MPD strains was tested against the C57BL/6J reference strain. Segmental germline DNA copy number gains and losses were evident in all strains (Figure 1). By using a set of stringent criteria (see Materials and Methods), 80 “high confidence” CNVs were identified. CNVs were detected on all 19 mouse autosomes (Figure 2). Changes on the X and Y chromosomes were not considered in this analysis because of lower probe density and greater mapping uncertainty for these regions in the current assembly. The segments vary in size (range, 21.4 kb to 2.0 Mb; mean, 271.5 kb) and number per strain (range, two CNVs per genome in C57BL/6Tac to 38 CNVs per genome in NOD/LtJ; mean, 22 CNVs per genome) (Table S1). As expected, no segments were identified in the C57BL/6J self–self hybridization. While the tiling-path design used in this study enables systematic detection of CNVs in genic and intergenic regions, we note that repeat-masking yields a subset of regions (e.g., the centromeres) with lower probe density, which could affect the CNV detection rate. Despite the lower probe coverage in these regions, several high confidence CNVs were detected in areas of relatively low probe coverage (Figure S1).

**Figure 3:**
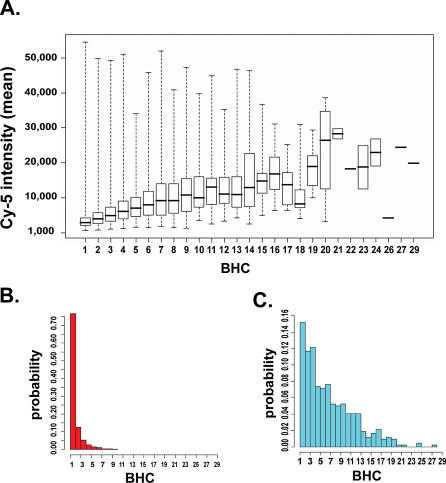
Relationship between Signal Intensity and Probe Uniqueness (A) Absolute signal intensity of the reference strain increases as a function of BHC (*p* < 10^−15^); 97.9% of autosomal probe sequences are present at single copy in the C57BL/6J genome*.* (B) Probes falling in “high confidence” CNVs are unique (1,005 of 1,313 probes with BHC = 1). (C) Probes falling in high signal CNVs are duplicated (356 of 420 probes with BHC > 1).

These CNVs were identified by comparing the ratio of signal intensity between the reference and test samples. Additional information can be extracted by analyzing the raw signal intensity of the reference (C57BL/6J) channel alone. For 94.6% of the probes on the array, the absolute signal intensity for C57BL/6J varies by less than one order of magnitude. Most of the high confidence CNV calls fall in these areas (Table S1 and Figure S2). Because the array design is based on the C57BL/6J reference genome (National Center for Biotechnology Information [NCBI] Build 34), sequences absent in C57BL/6J and present in other strains are not detected in this analysis. Segments amplified in C57BL/6J may appear as relative copy number loss in comparison strains or may be missed if these strains contain similar amplifications. To identify these duplicated regions, the average absolute signal intensity values for the reference strain were scanned using a statistical process control algorithm (see Materials and Methods). Sixty-seven high signal intensity locations were flagged, of which 13 overlapped CNVs (Table S2 and Figure S2). Twelve of these segments were also identified in an independent set of 20 CGH experiments using the same array design (unpublished data). To determine whether probe sequence redundancy might underlie the high reference signal in these regions, we searched the genome (NCBI Build 36) for nearly perfect matches to each probe using BLAT [9]. A measure of probe redundancy, BLAT hit count (BHC), was defined as the number of matches in which the sequence identity normalized to the length of the probe is greater than or equal to 0.90. There is a linear relationship (*p* < 10^−15^) between the BHC and the average signal of the reference channel across all 21 arrays (Figure 3). The BHC in regions of normal signal intensity (Figure 3, mean = 1.46) is significantly less than the BHC in regions with high reference signal (Figure 3, mean = 6.27). The strong association between BHC and signal intensity suggests that the high signal intensity segments are amplified regions in the C57BL/6J genome. Although their structure is clearly different than that of CNVs falling in areas of lower reference signal intensity, these segments are also sites of genomic variability between the MPD strains (Figure S2 and Table S2). This idea reinforces the recent report that the distribution of some human CNVs is not bimodal between populations but rather a continuous variable with properties of quantitative traits [10].

### PCR Validation of Oligo-aCGH Results

**Figure 4:**
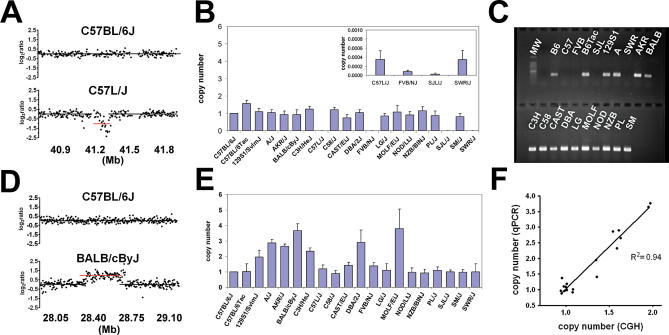
Validation of Copy Number Changes Identified by aCGH (A) Log_2_ ratio plot demonstrates a 109.2-kb segment of copy number loss on Chromosome 6 in C57L/J, compared to C57BL/6J. (B) qPCR using a primer/probe set in the altered region demonstrates normal copy number (normalized to a relative copy number of one in C57BL/6J) in unaffected strains and significantly reduced copy number in four affected strains (inset, zoom-in view of *y*-axis). (C) qPCR fails to generate an amplicon of expected size in the altered region from affected strains. B6, C57BL/6J reference strain. (D) Log_2_ ratio plot demonstrates a 473.7-kb segment of copy number gain on Chromosome 17 in BALB/cByJ compared to C57BL/6J. (E) qPCR demonstrates heterogeneity of copy number (normalized to a relative copy number of one in C57BL/6J) in this region among 20 strains. (F) Copy number estimates from aCGH and qPCR are highly concordant (*p* < 0.0001).

Quantitative PCR (qPCR) was used to validate several high confidence CNVs. Primer/probe assays were designed that are fully contained within CNVs. The results were normalized to segments that are not altered in any of the strains included in this study. Copy number assessed by qPCR was concordant with oligo-aCGH in unaffected strains (Figure 4). DNA from strains with copy number loss (e.g., Figure 4A) by oligo-aCGH yielded only background level amplification by PCR, implying that the target sequences for the assay were not present in these strains (Figure 4B). These results were confirmed using a second, independent qPCR assay within the same segment (unpublished data). Finally, no amplicon was generated in qualitative PCRs using a third set of primer pairs in this region (Figure 4C). Amplified segments (e.g., Figure 4D) detected by oligo-aCGH were confirmed by qPCR using the relative comparative threshold cycle (C_T_) method [11] (Figures 4E and S3). Regression analysis demonstrated a linear relationship between copy number determined by both platforms (Figure 4F) (*p* < 0.0001). All high confidence CNVs subjected to qPCR validation to date (*n* = 9) have been confirmed (Figures 4, S3, and S4).

### Relationship between CNV Profiles and Strain Histories

**Figure 5:**
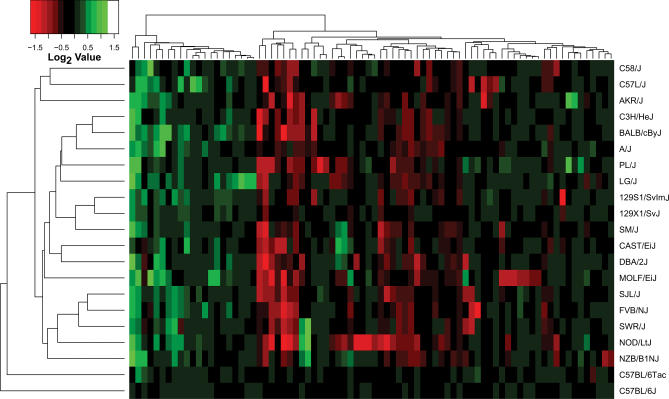
Heatmap Representation of Copy Number Changes in Mice Unsupervised clustering of segmental gains (green) and losses (red) yields a dendrogram that recapitulates features of the known genealogy of these strains. Clustering in the vertical axis demonstrates three clusters: segments amplified in most strains, segments reduced in most strains, and a third cluster containing either singleton CNVs or mixtures of amplifications and deletions.

Hierarchical clustering was performed to identify structure in the CNV profiles for these 21 strains. Unsupervised clustering of the 80 high confidence CNVs (Figure 5) illustrates three distinct groups of CNVs: segments deleted in more than one strain, segments amplified in more than one strain, and a third cluster containing singleton CNVs (CNVs occurring in only one strain) and “mixed” CNVs (CNVs that are both deleted and amplified, nine in total). The CNV profiles from replicate hybridizations were highly correlated (Figure S5), demonstrating the reproducibility of CNV detection by this platform. The cophenetic correlation of the dendrogram is 0.851, indicating that the distance matrix is appropriately represented as a tree. Unsupervised clustering by Pearson correlations between CNV profiles recapitulates some aspects of known mouse genealogy [12]. Strains 129X1/SvJ and 129S1/SvImJ stably cluster (bootstrap value = 0.80) in agreement with their known recent shared ancestry [13]. Swiss-derived strains FVB/NJ, SJL/J, and SWR/J cluster together and with another Swiss-derived strain, NOD/LtJ, although the bootstrap values (0.60 and 0.26, respectively) are not significant. Although C57BL/6J and C57BL/6Tac are very closely related, they do not cluster together. This is an artifact of the measurements being based on a C57BL/6J standard. When Euclidean distance is used as the metric, C57BL/6J and C57BL/6Tac group together, reflecting their genealogical relationship. The remainder of the tree lacks resolution, indicating that, for the most part, the relationships between strains can be considered a star phylogeny, with only a little structure at the tips of the tree. This is also consistent with the breeding history of the strains [12]. A single nucleotide polymorphism (SNP)-derived tree differs mainly in a strong cluster composed of only the wild-derived strains CAST/EiJ and MOLF/EiJ, possibly reflecting ascertainment bias in the SNP panel [14]. This acknowledged limitation of the current mouse SNP database [14] will be remedied as data emerge from the ongoing Mouse Genome Resequencing and SNP Discovery Project (http://www.niehs.nih.gov/crg/cprc.htm).

### Comparison of Low-Resolution and High-Resolution aCGH Analyses

A systematic comparison of the 80 CNVs in this report to previously described mouse CNVs uncovered 17 of 238 overlapping CNVs from one study [6] and three of 74 from another [7] (Table S4). A single CNV located between 63 Mb and 64 Mb on Chromosome 14 was identified in all three studies. This CNV is amplified in C57BL/6J in one report [7], deleted in 12 strains in the present study including FVB/NJ (the reference strain used in the previous study [7]), and deleted in five strains in another study [6]. It is likely that this CNV is an amplification in C57BL/6J, resulting in an apparent deletion in multiple strains with normal copy number in our study. This cross-study comparison was limited to the small number of strains overlapping between the current study and the prior reports and to those previously reported CNVs whose genome position in Build 34 could be determined (85.9% and 62.9% of CNVs, respectively) [6,7]. Differences in the analytical techniques and the reference genome used also contribute to the relatively low overlap between these studies.

The use of oligo-aCGH overcomes several limitations of the BAC array platform used in prior studies. Whereas CNV calls are frequently made on the basis of a single BAC hybridization event, 15 to 20 probes cover an interval of similar size on the 5-kb tiling-path oligo-aCGH platform used here. More important, the increased resolving power of oligo-aCGH can discern complex CNV structures such as adjacent amplifications and deletions (e.g., Chromosome 6 at 130.7 and 130.9 Mb) and identify smaller-sized CNVs, as have been found in the human genome [15]. Since our analytical approach was weighted to minimize false positives, our results are an underestimate of the total number of CNVs in the mouse genome. Using a relaxed set of criteria (i.e., called by process control and circular binary segmentation but not subjected to manual curation or qPCR validation), the number of potential CNVs detected by oligo-aCGH in these strains increases to 463 (unpublished data). Ongoing studies using higher probe density (e.g., 200-bp median spacing for an effective resolution of approximately 1 kb) are needed for validation of these apparent copy number changes.

### Segmental Duplications Colocalize with CNVs in the Mouse Genome

Although the complete set of mechanisms responsible for generating CNVs is unknown, previous studies have noted the enrichment of CNVs near segmental duplications in the human [3,4,16–18] and mouse [6] genomes. Segmental duplications are genomic regions of high sequence identity (greater than or equal to 90%) to more than one genomic locus and have been mapped in both the human [19] and mouse [20,21] genomes. They may mediate CNV genesis by acting as a substrate for nonallelic homologous recombination. A nonallelic homologous recombination event may result in amplification, deletion, inversion, or no copy number change. To test whether the nonrandom association between CNVs and segmental duplications is preserved in our high-resolution data, we determined the overlap of CNVs with segmental duplications (distances from CNV to nearest segmental duplication shown in Tables S1 and S2). Thirty-eight of 80 (47.5%; *p* < 0.001 by permutation testing) CNVs directly overlapped segmental duplications obtained from the Non-Human Segmental Duplication Database (http://projects.tcag.ca/xenodup/data.php) [21].

**Figure 6:**
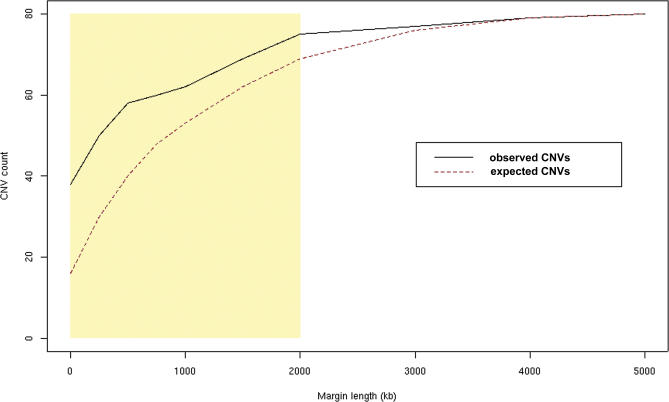
Relationship between Genomic Distance and Overlap between Segmental Duplications and CNVs The number of CNVs that overlap at least one segmental duplication was calculated for a range of margin sizes. At a margin size of zero (complete overlap with CNV), 38 of 80 observed CNVs overlap segmental duplications. The extent of overlap between CNVs and segmental duplications (black solid line) increases with margin size. The red dotted line (expected CNVs) indicates the colocalization of segmental duplications with randomly permuted genomic regions of lengths equal to the observed CNVs. Each point of the permuted data was calculated by determining the 95th percentile of the overlap counts. The association between CNVs and segmental duplications remains significant to the 2-Mb window size (*p* < 0.01) and is highlighted in the yellow rectangle.

We also tested a range of margin sizes, defined as the number of base pairs flanking a CNV in either direction, since a direct overlap between a CNV and segmental duplication may not be necessary for CNV genesis. The association remained significant (*p* < 0.01) up to 2 Mb, providing an estimate of the upper limit at which segmental duplications may affect CNVs (Figure 6). The high proportion of CNVs colocalizing with segmental duplications (94% at 2 Mb) can be interpreted as a bias in our CNV identification methodology or as support for a strong, almost necessary, role of segmental duplications in CNV generation. The distance between CNVs and segmental duplications is an important parameter of the mechanism driving segmental duplication–mediated CNV creation. Because some experimental approaches search for CNVs only in segmental duplication regions [18], it is also important to know the distribution of CNV–to–segmental duplication distances so that all potential CNV-containing regions can be screened.

Successful detection of these overlaps is dependent on both the precise definition of a segmental duplication (in this case, a length of 5 kb or greater with at least 90% sequence identity) and the accuracy of the segmental duplication database. Because the genomic coordinates of segmental duplications were determined with a previous build of the genome, we used liftOver (http://genome.ucsc.edu/cgi-bin/hgLiftOver) to map the coordinates to Build 34. In this process, 7,748 of 44,851 (17%) segmental duplications failed to remap. It is unlikely that a larger proportion of the 80 high confidence CNVs colocalizes with segmental duplications, because the majority of the unmapped segmental duplications were originally localized to sex chromosomes, which were excluded from our analysis.

### Gene Content of Mouse CNVs

We asked whether the gene content of CNVs is representative of the whole mouse genome. In the 80 high confidence CNVs reported here, 62 (77.5%) contain or overlap at least one gene. Genomic regions with lengths drawn from the distribution of detected CNVs were randomly selected, and the number of segments overlapping at least one gene was counted. The probability of 62 or more CNVs overlapping at least one gene each is 0.12 based on 1,000 randomizations, indicating that the gene content of these CNVs is not significantly different from the whole mouse genome.

We next asked if genes overlapping CNVs are enriched in Gene Ontology (GO) annotations. Several GO categories were significantly (*p* < 0.001) overrepresented in our mouse CNV data (Table 1). The term “carbohydrate binding” is overrepresented in concordance with a previous GO analysis of mouse CNVs [22]. We also found several annotations to be overrepresented that were previously reported to be significantly underrepresented: G protein–coupled receptor activity, rhodopsin-like receptor activity, and olfactory receptor activity. The discrepancy in GO enrichment may be attributable to the fact that the prior analysis was based on data covering fewer strains. Several terms overrepresented in our analysis of mouse CNVs were also found to be overrepresented in an analysis of human CNV data (e.g., transmembrane receptor activity, olfactory receptor activity) [22].

**Table 1:**
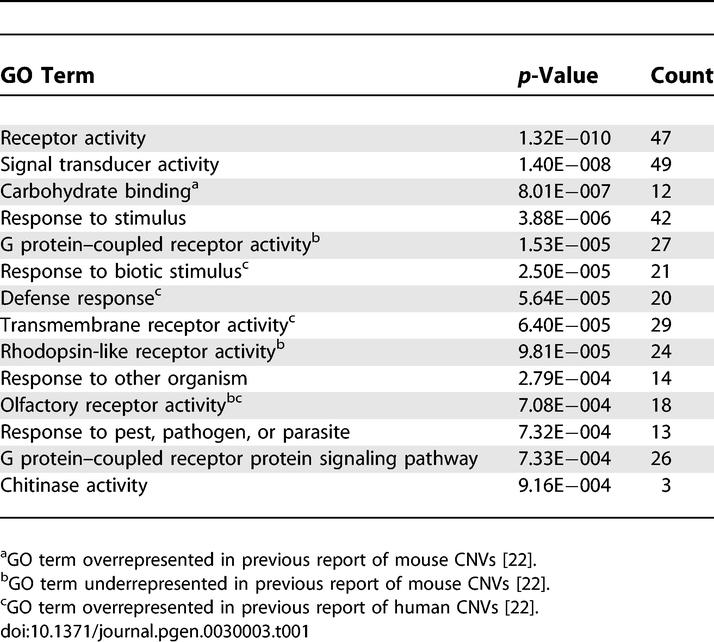
GO Categories Significantly Overrepresented in Mouse CNVs

The human orthologs of several of the genes contained within the mouse CNVs identified in this study *(Immp2l*, *Birc1*, *Cfh*, *Sirpb1*, and *Btbd9)* have had reported germline copy number polymorphisms (http://projects.tcag.ca/variation). Conservation of CNVs across species suggests that selective pressure may drive acquisition or retention of specific gene dosage alterations. One of these genes *(CFH)* is of particular interest because, in addition to germline CNV, an SNP (Y402H) was recently shown to account for a substantial proportion of the heritable risk for age-related macular degeneration in humans [23–25].

Two high confidence mouse CNVs reported here include portions of the *Klra* gene cluster on Chromosome 6. The large *Klra* (Ly49) family of C-type lectin transmembrane proteins are the functional analogs of the human natural killer cell immunoglobulin-related receptors (KIR). In the current genome assembly (Build 36), these two segments on Chromosome 6 contain 12 *Klra* family members, including *Klra8*. Concordant with our oligo-aCGH results, sequence analysis in BALB/c and DBA/2 mice demonstrated loss of *Klra8* (Ly49H) [26], a critical resistance factor for mouse cytomegalovirus infection [27,28]. Our data predict that AKR/J, C3H/HeJ, and PL/J should also be susceptible to mouse cytomegalovirus infection and that the remaining strains should be resistant. Similarly, the intelectins are intestinal epithelial cell surface proteins involved in innate immunity to parasitic infection. We found copy number increase in the locus encoding intelectin-1 *(Intlna)* on Chromosome 1 in several strains, including BALB/cByJ. These results confirm previous reports of intelectin gene duplication in BALB/c and an associated increase in resistance to *Trichinella* infection compared to C57BL/6 [29]. These examples independently validate this CNV discovery platform and provide proof of principle that CNVs may underlie many phenotypic differences between mouse strains.

A recent comprehensive study of copy number variation in the human genome [36] revealed many findings concordant with our study of the mouse genome. Using SNP arrays and CGH with a large insert clone array, extensive CNV was detected in apparently normal individuals from the HapMap collection. The human CNVs are associated with segmental duplications and contain genes enriched for sensory perception GO categories. Taken together, these studies demonstrate the extent and significance of CNV in the mammalian genome.

## Materials and Methods

### Oligonucleotide array construction.

A tiling-path CGH array for whole-genome analysis in mouse (NCBI Build 34) was designed and constructed by NimbleGen Systems (http://www.nimblegen.com). Probes were selected with a minimum probe spacing of 4.5 kb, and the resulting array had a median probe spacing of 5.2 kb. Probes were synthesized using an isothermal format (T_m_ = 76 °C) and varied in length from 45 to 75 bp.

### Sample processing.

Male mice aged 8 to 10 wk were obtained from the research colony at The Jackson Laboratory or Taconic (http://www.taconic.com). Genomic DNA was prepared from spleen and tail specimens (DNeasy; Qiagen, http://www.qiagen.com). Unamplified genomic DNA (1 μg) was labeled with Cy3 (test strains) or Cy5 (reference strain, C57BL/6J), and hybridizations were performed by NimbleGen Systems in a two-color format according to Selzer et al. [30].

### Statistical analysis.

Each array data set was imported into R [31] for normalization. The normalize.qspline method from the Bioconductor package [32] was used to normalize the signal intensities of the sample versus reference. A Statistical Process Control algorithm (X. Li, A. Allred, M. Walter, R. Ries, T. Ley, W. Shannon, unpublished data) was used to flag log_2_ (test/reference) values that deviate significantly from baseline. The segment boundaries were defined using circular binary segmentation [33] across the flagged region. A set of conservative criteria (five or more probes in a segment, mean amplitude of log_2_ shift across segment = ±0.5) were used to define the final set of high confidence CNV calls. CNV profiles and strains were clustered with hierarchical clustering using average linkage and the Pearson correlation coefficient as the distance. The probe density was computed by counting the number of probes within each 500-kb window of the genome. The BLAT hit count was defined as the number of matches in which the probe sequence identity × length of matching sequence/length of the probe was greater than or equal to 0.90. Gene annotation and overlap were determined using Ensembl Genebuild July 2005, database version 32.34 [34]. Gene enrichment was tested by randomly selecting a segment length from the distribution of high confidence CNV lengths, randomly selecting a valid chromosomal location, and determining if the segment overlapped at least one gene. A similar approach was used to test for association between CNVs and segmental duplications. GO analysis was performed using DAVID Bioinformatic Resources 2006 (http://david.abcc.ncifcrf.gov/home.jsp) [35]. Comparison of CNVs to previous publications was assessed by remapping CNVs from the appropriate NCBI assembly version (Builds 30 and 32) using liftOver and determining the overlap of reported CNVs.

### PCR validation.

To validate CNVs detected by oligo-aCGH, qPCR assays were used to measure copy number in altered regions relative to a control region of invariant copy number across all 21 strains (selected based on aCGH profiles). Relative copy numbers were determined by real-time PCR (qPCR) using TaqMan detection chemistry and the ABI Prism 7300 Sequence Detection System (Applied Biosystems, http://www.appliedbiosystems.org). Primers and TaqMan probes were designed using ABI Primer Express Software (version 2.0) and the NCBI Mouse Build 34 (primer and probe sequences provided in Table S3). All probes were dual-labeled with 6-carboxyfluorescein and 6-carboxytetramethyl-rhodamine. Each assay was performed in triplicate using 25-μl reactions containing 12.5 μl of TaqMan 2× PCR master mix (Applied Biosystems), 280 nM TaqMan probe, 900 nM concentration of forward and reverse primer, and 100 ng of genomic DNA. Amplification was performed according to the following conditions: one cycle at 50 °C for 2 min, one cycle at 95 °C for 10 min, and 50 cycles at 95 °C for 15 s and 60 °C for 1 min. Experiments were performed on the test and control primers to verify comparable efficiency in amplification prior to analysis of copy number in the strains. The C_T_ method was used for quantification of copy number in the test strains relative to the reference strain (C57BL/6J). The C_T_ values for each set of triplicates were averaged and were normalized against the control primer. The relative copy number for each strain was calculated as 2^− (normalized C^T^ for the test strain − normalized C^T^ for C57BL/6)^. A linear regression model was used to compare the copy number estimates from aCGH and qPCR.

CNVs were also assessed by non-qPCR. Amplification reactions contained 20 μl of Jumpstart Ready Mix Taq (Sigma, http://www.sigmaaldrich.com), 2.5 ng of each primer, and 100 ng of genomic DNA in a final volume of 40 μl. Amplifications were performed on a Gene Amp 9700 (PE Applied Biosystems) at standard conditions for 35 cycles, and the product was run on a 1.5% agarose gel, stained with ethidium bromide, and visualized on a UV transilluminator.

## Supporting Information

Figure S1 Relationship between CNVs and Genome-Wide Probe Coverage by Oligo-aCGH Segmental gains and losses are shown to the left of each chromosome (as in Figure 2). The probe density in 500-kb windows across the genome is shown to the right of each ideogram.
(1.3MB TIF).

Figure S2 Detection of CNV Is Affected by Reference DNA Signal Intensity (A) CNV in a region of normal reference DNA signal on Chromosome 6. The mean signal intensity (*n* = 21 arrays) for the reference sample (C57BL/6J) ranges from 1 to 10,000 across this region (inset).
(B) CNV in a region of high reference DNA signal on Chromosome 8. The segment boundaries coincide with a region of high mean signal intensity for the reference DNA (inset).
(4.2 MB TIF)

Figure S3 Additional Validation of aCGH Results Using qPCR: Part I Log_2_ plot and qPCR data from (A) an 82.8-kb amplified region on Chromosome 18 in AKR/J and PL/J, (B) a 93.8-kb deleted region on Chromosome 3 in NOD/LtJ, (C) a 51.8-kb deleted region on Chromosome 19 in CAST/EiJ, FVB/NJ, and MOLF/EiJ, and (D) a 110.6-kb segment deleted on Chromosome 11 in MOLF/EiJ (inset, zoom-in view of *y*-axis). The start site of each CNV is shown next to the log_2_ plots.
(1.7 MB TIF)

Figure S4 Additional Validation of aCGH Results Using qPCR: Part II Log_2_ plot and qPCR data from (E) a 47.0-kb segment deleted on Chromosome 1 in BALB/cByJ, C3H/HeJ, and PL/J, (F) a 1.1-Mb segment deleted on Chromosome 4 in MOLF/EiJ, CAST/EiJ, and SM/J, and (G) a 42.0-kb segment on Chromosome 6 amplified in SWR/J and deleted in C3H/HeJ and PL/J (inset, zoom-in view of *y*-axis).
(3.6 MB TIF)

Figure S5 Unsupervised Clustering of CNVs in 21 MPD Strains with Replicates Ten replicates were studied, including repeat hybridizations with the same sample or a different tissue from the same animal (spleen versus tail DNA). The Pearson correlation coefficient was computed for the log_2_ signal for all probes for each pairwise chip–chip comparison. The probe signal correlation coefficients are low since there is no global normalization among arrays. However, there is a strong correlation among the CNV profiles of replicate samples (mean correlation, 0.92; range, 0.84 to 0.96). Replicate number is indicated in parentheses. Sp, spleen.
(3.9 MB TIF)

Table S1 Segmental Variation in Areas Not Amplified in Reference Strain (C57BL/6J) (102 KB XLS)

Table S2 Segmental Variation in Regions Amplified in Reference Strain (C57BL/6J) (74 KB XLS)

Table S3 Primers Used for CNV Validation (15 KB XLS)

Table S4 Comparison of CNVs in This Study with Previous Publications (65 KB XLS)

### Accession Numbers

Primary data from the oligo-aCGH experiments can be found in the NCBI Gene Expression Omnibus (GEO; http://www.ncbi.nlm.nih.gov/geo) under accession number GSE5805.

## Abbreviations

BHC: BLAT hit count
CNV: copy number variant
C_T_: comparative threshold cycle
GO: Gene Ontology
MPD: Mouse Phenome Database
NCBI: National Center for Biotechnology Information
oligo-aCGH: long oligonucleotide array comparative genomic hybridization
qPCR: quantitative PCR
SNP: single nucleotide polymorphism

## References

[pgen-0030003-b001] Wiltshire T, Pletcher MT, Batalov S, Barnes SW, Tarantino LM (2003). Genome-wide single-nucleotide polymorphism analysis defines haplotype patterns in mouse. Proc Natl Acad Sci U S A.

[pgen-0030003-b002] Yalcin B, Fullerton J, Miller S, Keays DA, Brady S (2004). Unexpected complexity in the haplotypes of commonly used inbred strains of laboratory mice. Proc Natl Acad Sci U S A.

[pgen-0030003-b003] Sebat J, Lakshmi B, Troge J, Alexander J, Young J (2004). Large-scale copy number polymorphism in the human genome. Science.

[pgen-0030003-b004] Iafrate AJ, Feuk L, Rivera MN, Listewnik ML, Donahoe PK (2004). Detection of large-scale variation in the human genome. Nat Genet.

[pgen-0030003-b005] Perry GH, Tchinda J, McGrath SD, Zhang J, Picker SR (2006). Hotspots for copy number variation in chimpanzees and humans. Proc Natl Acad Sci U S A.

[pgen-0030003-b006] Li J, Jiang T, Mao J, Balmain A, Peterson L (2004). Genomic segmental polymorphisms in inbred mouse strains. Nat Genet.

[pgen-0030003-b007] Snijders A, Nowak N, Huey B, Fridlyand J, Law S (2005). Mapping segmental and sequence variations among laboratory mice using BAC array CGH. Genome Res.

[pgen-0030003-b008] Sharp AJ, Hansen S, Selzer RR, Cheng Z, Regan R (2006). Discovery of previously unidentified genomic disorders from the duplication architecture of the human genome. Nat Genet.

[pgen-0030003-b009] Kent W (2002). BLAT—The BLAST-like alignment tool. Genome Res.

[pgen-0030003-b010] Locke DP, Sharp AJ, McCarroll SA, McGrath SD, Newman TL (2006). Linkage disequilibrium and heritability of CNPs within duplicated regions of the human genome. Am J Hum Genet.

[pgen-0030003-b011] Ginzinger DG, Godfrey TE, Nigro J, Moore DH, Suzuki S (2000). Measurement of DNA copy number at microsatellite loci using quantitative PCR analysis. Cancer Res.

[pgen-0030003-b012] Beck JA, Lloyd S, Hafezparast M, Lennon-Pierce M, Eppig JT (2000). Genealogies of mouse inbred strains. Nat Genet.

[pgen-0030003-b013] Threadgill D, Yee D, Matin A, Nadeau J, Magnuson T (1997). Genealogy of the 129 inbred strains: 129/SvJ is a contaminated inbred strain. Mamm Genome.

[pgen-0030003-b014] Pletcher MT, McClurg P, Batalov S, Su AI, Barnes SW (2004). Use of a dense single nucleotide polymorphism map for in silico mapping in the mouse. PLoS Biol.

[pgen-0030003-b015] Conrad DF, Andrews TD, Carter NP, Hurles ME, Pritchard JK (2006). A high-resolution survey of deletion polymorphism in the human genome. Nat Genet.

[pgen-0030003-b016] Tuzun E, Sharp AJ, Bailey JA, Kaul R, Morrison VA (2005). Fine-scale structural variation of the human genome. Nat Genet.

[pgen-0030003-b017] Cheung J, Estivill X, Khaja R, MacDonald J, Lau K (2003). Genome-wide detection of segmental duplications and potential assembly errors in the human genome sequence. Genome Biol.

[pgen-0030003-b018] Sharp AJ, Locke DP, McGrath SD, Cheng Z, Bailey JA (2005). Segmental duplications and copy-number variation in the human genome. Am J Hum Genet.

[pgen-0030003-b019] Bailey J, Gu Z, Clark R, Reinert K, Samonte R (2002). Recent segmental duplications in the human genome. Science.

[pgen-0030003-b020] Bailey J, Church D, Ventura M, Rocchi M, Eichler E (2004). Analysis of segmental duplications and genome assembly in the mouse. Genome Res.

[pgen-0030003-b021] Cheung J, Wilson M, Zhang J, Khaja R, MacDonald J (2003). Recent segmental and gene duplications in the mouse genome. Genome Biol.

[pgen-0030003-b022] Nguyen DQ, Webber C, Ponting CP (2006). Bias of selection on human copy-number variants. PLoS Genet.

[pgen-0030003-b023] Klein RJ, Zeiss C, Chew EY, Tsai JY, Sackler RS (2005). Complement factor H polymorphism in age-related macular degeneration. Science.

[pgen-0030003-b024] Edwards AO, Ritter R, Abel KJ, Manning A, Panhuysen C (2005). Complement factor H polymorphism and age-related macular degeneration. Science.

[pgen-0030003-b025] Haines JL, Hauser MA, Schmidt S, Scott WK, Olson LM (2005). Complement factor H variant increases the risk of age-related macular degeneration. Science.

[pgen-0030003-b026] Anderson SK, Dewar K, Goulet ML, Leveque G, Makrigiannis AP (2005). Complete elucidation of a minimal class I MHC natural killer cell receptor haplotype. Genes Immun.

[pgen-0030003-b027] Lee SH, Girard S, Macina D, Busa M, Zafer A (2001). Susceptibility to mouse cytomegalovirus is associated with deletion of an activating natural killer cell receptor of the C-type lectin superfamily. Nat Genet.

[pgen-0030003-b028] Brown MG, Dokun AO, Heusel JW, Smith HR, Beckman DL (2001). Vital involvement of a natural killer cell activation receptor in resistance to viral infection. Science.

[pgen-0030003-b029] Pemberton AD, Knight PA, Gamble J, Colledge WH, Lee JK (2004). Innate BALB/c enteric epithelial responses to *Trichinella spiralis:* Inducible expression of a novel goblet cell lectin, intelectin-2, and its natural deletion in C57BL/10 mice. J Immunol.

[pgen-0030003-b030] Selzer RR, Richmond TA, Pofahl NJ, Green RD, Eis PS (2005). Analysis of chromosome breakpoints in neuroblastoma at sub-kilobase resolution using fine-tiling oligonucleotide array CGH. Genes Chromosomes Cancer.

[pgen-0030003-b031] Ihaka R, Gentleman RC (1996). A language for data analysis and graphics. J Comp Graph Statist.

[pgen-0030003-b032] Gentleman RC, Carey VJ, Bates DM, Bolstad B, Dettling M (2004). Bioconductor: open software development for computational biology and bioinformatics. Genome Biol.

[pgen-0030003-b033] Olshen AB, Venkatraman ES, Lucito R, Wigler M (2004). Circular binary segmentation for the analysis of array-based DNA copy number data. Biostatistics.

[pgen-0030003-b034] Hubbard T, Andrews D, Caccamo M, Cameron G, Chen Y (2005). Ensembl 2005. Nucl Acids Res.

[pgen-0030003-b035] Dennis G, Sherman BT, Hosack DA, Yang J, Gao W (2003). DAVID: Database for annotation, visualization, and integrated discovery. Genome Biol.

[pgen-0030003-b036] Redon R, Ishikawa S, Fitch KR, Feuk L, Perry GH (2006). Global variation in copy number in the human genome. Nature.

